# Circulating human cytomegalovirus-encoded HCMV-miR-US4-1 as an indicator for predicting the efficacy of IFNα treatment in chronic hepatitis B patients

**DOI:** 10.1038/srep23007

**Published:** 2016-03-10

**Authors:** Yi Pan, Nan Wang, Zhenxian Zhou, Hongwei Liang, Chaoyun Pan, Dihan Zhu, Fenyong Liu, Chen-Yu Zhang, Yujing Zhang, Ke Zen

**Affiliations:** 1 State Key Laboratory of Pharmaceutical Biotechnology, Nanjing Advanced Institute for Life Sciences (NAILS), Nanjing University, 22 Hankou Road, Nanjing, Jiangsu 210093, China; 2 Jiangsu Engineering Research Center for microRNA Biology and Biotechnology, School of Life Sciences, Nanjing University, Nanjing 210093, China; 3 Clinical Laboratory, Nanjing Second Hospital, Nanjing 210003, China; 4 Department of Virology, University of California School of Public Health, Berkeley, CA 94720, USA

## Abstract

The efficacy of interferon α (IFNα) therapy for chronic hepatitis B (CHB) patients is about 40% and often associates with adverse side-effects, thus identification of an easy accessible biomarker that can predict the outcome of IFNα treatment for individual CHB patients would be greatly helpful. Recent reports by us and others show that microRNAs encoded by human cytomegalovirus (HCMV) were readily detected in human serum and can interfere with lymphocyte responses required by IFNα therapeutic effect. We thus postulate that differential expression profile of serum HCMV miRNAs in CHB patients may serve as indicator to predict the efficacy of IFNα treatment for CHB patients. Blood was drawn from 56 individual CHB patients prior to IFNα treatment. By quantifying 13 HCMV miRNAs in serum samples, we found that the levels of HCMV-miR-US4-1 and HCMV-miR-UL-148D were significantly higher in IFNα-responsive group than in IFNα-non-responsive group. In a prospective study of 96 new CHB patients, serum level of HCMV-miR-US4-1 alone classified those who were and were not responsive to IFN-α treatment with correct rate of 84.00% and 71.74%, respectively. In conclusion, our results demonstrate that serum HCMV-miR-US4-1 can serve as a novel biomarker for predicting the outcome of IFNα treatment in CHB patients.

Hepatitis B is an infectious disease of viral origin that is particularly prevalent in undeveloped areas of Asia and Africa. It is estimated to affect over 350 million people worldwide, with a mortality rate of over 1.2 million deaths per year due to acute or chronic hepatitis B (CHB) infection. There are two main classes of antiviral drugs for the treatment of CHB: interferon α (IFNα) and nucleoside analogs. As the only approved treatment for CHB infection in most countries, IFNα is widely used for the treatment of acute or chronic HBV infection. However, IFNα treatment can induce a long-term sustained remission in only 25% to 40% of CHB patients. In addition, a wide range of adverse side effects, such as severe hypertransaminasemia, depression, febrile convulsions and relapsing episodes of epistaxis associated with normal values of platelets and prothrombin time, are associated with IFNα treatment. Thus, it would be extremely helpful to have an effective and convenient molecular biomarker to predict the efficacy of IFNα treatment for individual CHB patients prior to treatment. Although previous studies have shown that the response to IFNα is dependent upon the HBV genotype with which the patient is infected^1^,^2^,^3^, there is currently no reliable and applicable biomarker that can predict the outcome of IFNα treatment in HBV patients.

MicroRNAs (miRNAs) are a class of small (19–23 nucleotides), conserved, non-coding RNA molecules that play a role in gene regulation at the posttranscriptional level^4^,^5^,^6^. Previous studies by our laboratory^7^,^8^ and others^9^,^10^,^11^ have shown that miRNAs are highly stable in plasma and serum and may serve as blood-based biomarkers in different cancers and other diseases. Interestingly, miRNAs encoded by infectious viruses have been also detected in human blood samples^12^,^13^,^14^. Human cytomegalovirus (HCMV) is a ubiquitous herpesvirus that can infect up to 90% of the adult population and persist for the lifetime of the host^15^,^16^. During productive infection, HCMV expresses at least 14 miRNAs that target genes relevant for viral proliferation and evasion of the immune response^17^,^18^. Infection by HCMV can modulate the host immune response and influence the infection by other virus and bacteria. A recent study by Li *et al.*^13^ showed that levels of the HCMV-encoded miRNA, HCMV-miR-UL112, were independently associated with an increased risk of essential hypertension.

In the present study, we hypothesize that there is a specific profile of serum miRNAs encoded by HCMV that can be used as a fingerprint to predict the efficacy of IFNα treatment in CHB patients. To test this hypothesis, we screened HCMV miRNAs by qRT-PCR assays in the serum of 28 CHB patients who were responsive to IFNα treatment and 28 CHB patients who were not responsive to IFNα treatment. HCMV-miR-US-1 and HCMV-miR-UL-148D were found to be differentially expressed in these two groups of CHB patients. A perspective study using serum samples from 96 new, independent CHB patients further confirmed that HCMV-miR-US4-1 alone could serve as an accurate biomarker for predicting the efficacy of IFNα treatment in CHB patients.

## Results

### Experimental design

**Figure 1:**
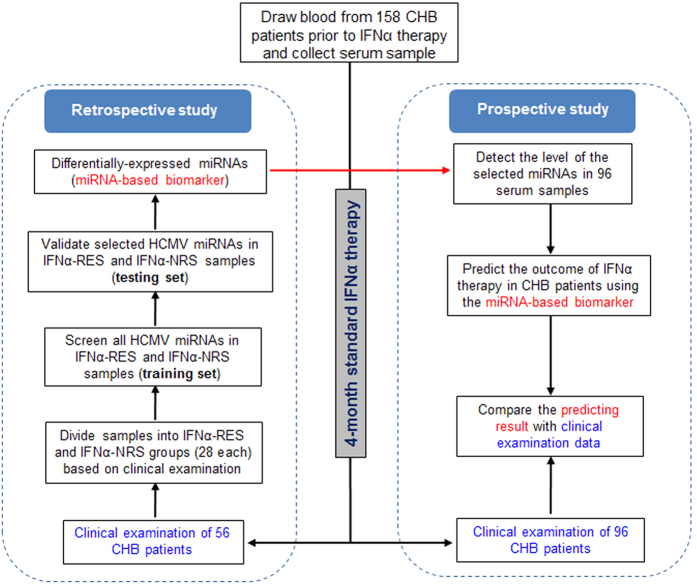
Overview of the experimental design.

As shown in the experimental flow chart (Fig. 1), we designed two main steps. Step 1 was to identify HCMV-encoded miRNAs in human serum samples that could serve as potential biomarkers for predicting the efficacy of IFNα therapy for CHB patients. Step 2 was to directly apply the identified HCMV miRNA-based biomarker to new CHB patients to test its accuracy in predicting the efficacy of IFNα therapy. In step 1, 56 serum samples from CHB patients prior to IFNα therapy were randomly assigned into training (20 patients) and testing (36 patients) groups. Following the 4-month course of IFNα treatment, the clinical diagnosis confirmed the training and testing sets composed of 20 and 36 IFNα responsive (INFα-RES) and non-responsive (INFα-NRS) patients, respectively. The training set and testing set each had 10 INFα-RES and 18 INFα-NRS patients. In step 2, serum samples were obtained from 96 new CHB patients prior to the IFNα therapy, and the efficacy of IFNα therapy in these patients was predicted using the identified HCMV miRNA-based biomarkers. The outcome of IFNα therapy in these CHB patients was determined by blood HBV-DNA titer examination in peripheral blood samples. If the blood HBV-DNA titer after treatment is lower than 500 copy/ml or 10% of the blood HBV-DNA titer before treatment, the patients are considered as INFα-RES. If not, they are considered as INFα-NRS. The information (name, age, sex, HBV-DNA, adjuvant therapy, ALT, AST, GTP, ALP, albumin, globulin, HBsAg, anti-HBc, leucocytes, hemoglobin, platelets, α-fetoprotein, and HBV-genotype) of the patients in INFα-RES group and INFα-NRS group were detailed in Supplementary Tables S1 and S2, respectively. All of the patients had been diagnosed to be negative for any other disease except for hepatitis B infection, and all the patients displayed no hepatic fibrosis. HCMV seropositivity assay showed that all the patients in the training set (28 each from INFα-RES or INFα-NRS group) were HCMV IgG positive but HCMV IgM negative except one patient from INFα-NRS group (Supplementary Fig. S1a). However, qRT-PCR assay indicated that 18 of 28 plasma samples from INFα-NRS group were HCMV DNA positive, while only 8 of 28 plasma samples from INFα-RES patients were HCMV DNA positive (64.3% versus 28.6%, P = 0.015; Supplementary Fig. S1b). INFα-NRS group also displayed a higher HCMV DNA titer than INFα-RES group (1475 versus 28 copies per 1 mL plasma, P = 0.017; Supplementary Fig. S1c). By comparing the efficacy predicted by the HCMV miRNA-based biomarker(s) with the outcome determined by the final clinical diagnosis, we would obtain the accuracy of the identified HCMV miRNA-based biomarker as a biomarker in predicting the efficacy of IFNα therapy for individual CHB patients.

### Differential expression of HCMV-encoded miRNAs in serum from HBV patients who were IFNα treatment responsive or non-responsive

**Figure 2:**
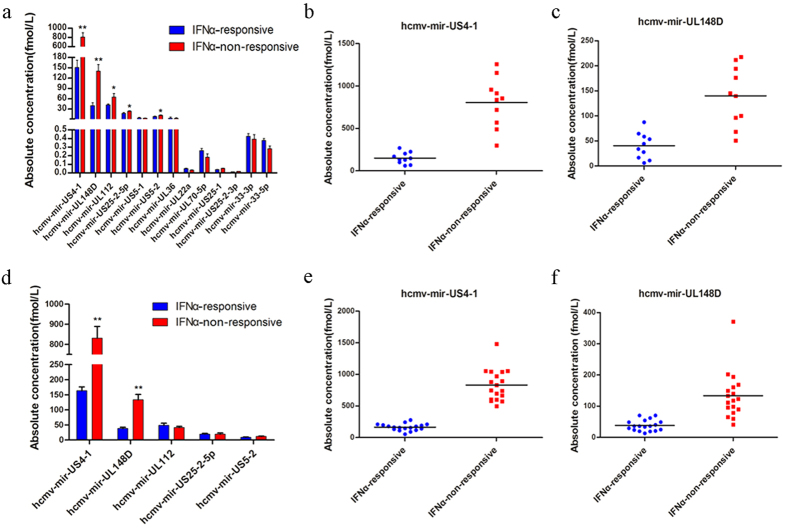
Serum levels of HCMV-encoded miRNAs in CHB patients arranged into training and validation sets. (**a**) Absolute concentrations of 13 HCMV-encoded miRNAs in training set samples (n = 10). (**b**) Scatter diagram of the absolute concentration of HCMV-miR-US4-1 in the samples of training set (*P* = 2.13 × 10^−6^). (**c**) Scatter diagram of the absolute concentration of HCMV-miR-UL148D in the training set samples (*P* = 1.3 × 10^−4^). (**d**) Absolute concentration of HCMV-miR-US4-1 and HCMV-miR-UL148D in the samples of testing set (n = 18). (**e**) Scatter diagram of the absolute concentration of HCMV-miR-US4-1 in the testing set (*P* = 6.65 × 10^−13^). (**f**) Scatter diagram of the absolute concentration of HCMV-miR-148D in the testing set (*P* = 7.26 × 10^−6^). The data are presented as the mean ± SEM. Horizontal lines indicate medians *P* values calculated by a 2-sided Student *t-*test. ^*^P < 0.05; ^**^P < 0.01.

With TaqMan probe-based qRT-PCR, we detected the expression profile of 13 HCMV miRNAs in serum samples from CHB patients in the training set. All CHB sera contained considerable amounts of HCMV miRNAs, suggesting that all CHB patients were HCMV-infected. This result is in agreement with the HCMV antigen screening, which indicated that all serum samples were HCMV IgG positive (Supplementary Fig. S1a). As shown in Fig. 2a, HCMV-miR-US4-1, HCMV-miR-UL-148D, HCMV-miR-UL112, HCMV-miR-US5-1 and HCMV-miR-US-5-2 were differentially expressed in serum samples from CHB patients in INFα-RES and INFα-NRS groups. The absolute level of HCMV-miR-US4-1 (Fig. 2b) and HCMV-miR-UL148D (Fig. 2c) in the INFα-NRS group was 5.2- and 3.0-fold greater, respectively, than that of the INFα-RES group. The significantly higher level of serum HCMV-miR-US4-1 and HCMV-miR-UL148D in CHB patients of INFα-NRS group, compared with the patients of INFα-RES group, was further validated using the serum samples from the testing sets. As shown in Fig. 2d, the serum levels of HCMV-miR-US4-1 and HCMV-miR-UL148D in CHB patients of INFα-NRS group were 5.4- and 3.3-fold greater, respectively, than that of CHB patients in the INFα-RES group. However, the serum levels of HCMV-miR-UL112, HCMV-miR-US5-1 and HCMV-miR-US-5-2 showed no significant difference between patients in the INFα-NRS and the INFα-RES groups. The qRT-PCR assay using the samples from the testing sets confirmed that CHB patients in the INFα-NRS group had a significantly higher serum level of HCMV-miR-US4-1 (Fig. 2e) and HCMV-miR-UL148D (Fig. 2f) than did CHB patients in the INFα-RES group. The detailed analysis of absolute levels of HCMV-miR-US4-1 and HCMV-miR-UL148D in the two groups of CHB patients from the training and testing sets was shown in Supplementary Tables S3 and S4, respectively. We also detected the expression of HCMV-miR-US4-1 in the serum samples from CHB patients after IFNα treatment. As shown in Supplementary Fig. S2, the absolute level of HCMV-miR-US4-1 in the INFα-NRS group was more than 5-fold higher than that in the INFα-RES group. Interestingly, there was no significant decrease of the absolute level of HCMV-miR-US4-1 in either INFα-RES or INFα-NRS groups after IFNα treatment. The main characteristics of these patients were shown in Supplementary Table S5.

### Serum HCMV-miR-US4-1 and HCMV-miR-UL148D allow discrimination of CHB patients in the IFNα responsive group from CHB patients in the IFNα non-responsive group

**Figure 3:**
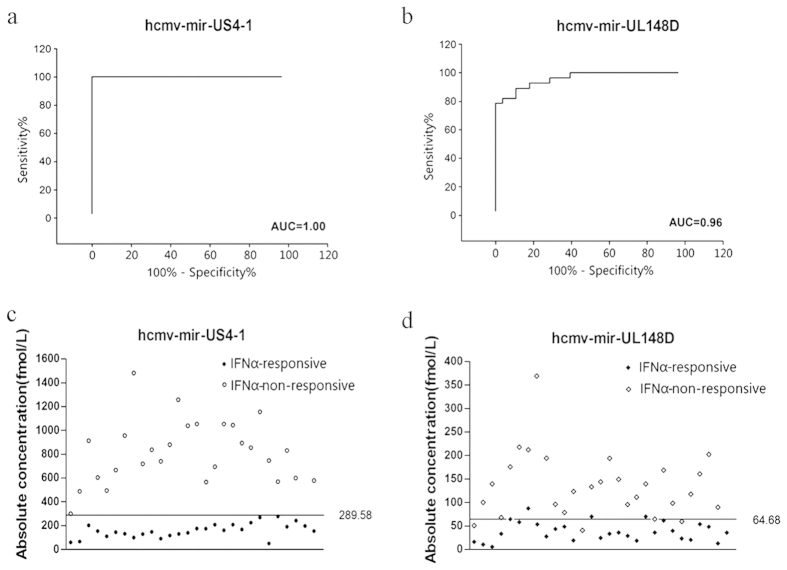
ROC curve analysis and cut off value among the training set and the validation set by HCMV-miR-US4-1 and HCMV-miR-UL-148D. (**a**) ROC curve of HCMV-miR-US4-1 as biomarker (AUC = 1.00). (**b**) ROC curve of HCMV-miR-UL-148D as biomarker (AUC = 0.96). (**c**) A scatter diagram showing the absolute concentration of HCMV-miR-US4-1 with the cutoff point at 289.58 fmol/L. (**d**) A scatter diagram displaying the absolute concentration of HCMV-miR-UL148D with the cutoff point at 64.68 fmol/L.

We next analyzed whether HCMV-miR-US4-1 and HCMV-miR-UL148D had potential to be signatures for separating CHB patients who were response to IFNα treatment from CHB patients who were non-responsive to IFNα treatment. Using the 5% and 95% reference interval of each miRNA expression value as risk scores, we constructed ROC curves and estimated the sensitivity and specificity for prediction. When HCMV-miR-US4-1 or HCMV-miR-UL148D was used as a signature to separate the IFNα-RES group from the INFα-NRS group of CHB patients, the AUC was 1.00 (sensitivity: 100%; specificity: 100%) (Fig. 3a) or 0.96 (sensitivity: 89.28%; specificity: 89.28%) (Fig. 3b). We then used each individual HCMV-encoded miRNA to construct a biomarker by a risk-score method. The patients in both the training and validation sets were ranked according to their risk scores and divided into the IFNα-RES group or the INFα-NRS group using the median risk score as the cutoff point. As shown in Fig. 3c,d, HCMV-miR-US4-1 or HCMV-miR-UL148D-based biomarkers could clearly separate CHB patients of the IFNα-RES group from the IFNα-NRS group, with zero and only three out of 56 cases being misclassified, respectively.

### As an indicator, serum HCMV-miR-US4-1 accurately predicts the efficacy of IFNα therapy for CHB patients

**Figure 4:**
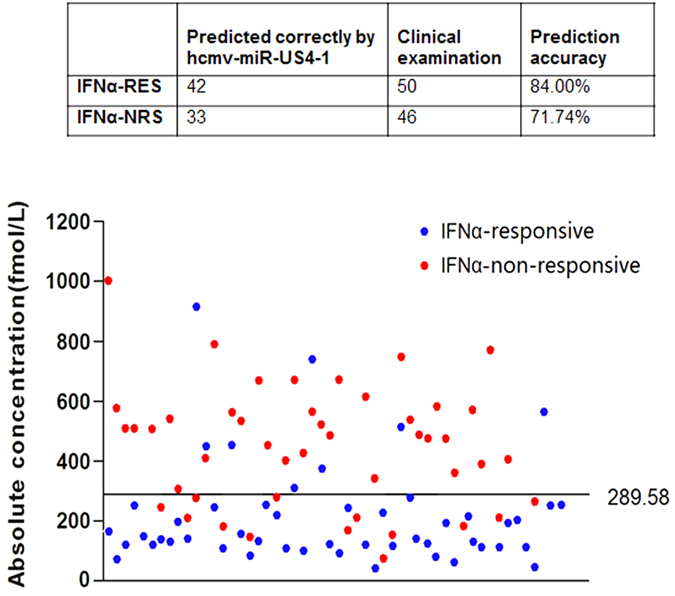
A perspective study of the accuracy of serum HCMV-miR-US4-1 levels as a biomarker to predict the outcome of IFNα therapy for individual CHB patients. (**a**) A box showing the sensitivity and specificity of the serum HCMV-miR-US4-1-based biomarker. (**b**) A scatter diagram showing the accuracy of serum HCMV-miR-US4-1 levels as a biomarker for predicting the efficacy of IFNα therapy for individual CHB patients.

The accuracy of HCMV-miR-US4-1 as an indicator for the efficacy of IFNα therapy for CHB patients was further tested using serum samples from 96 new CHB patients in a perspective fashion. First, the serum samples were obtained from each CHB patient prior to IFNα therapy and immediately assessed for levels of HCMV-miR-US4-1. The same risk-score formula and cutoff point obtained for HCMV-miR-US4-1 from the training/validation sets were directly applied to these new CHB patients, and the efficacy of IFNα therapy for individual CHB patients was predicted. Second, following IFNα therapy, patients were subjected to a clinical diagnosis and determined to be IFNα responsive or IFNα non-responsive based on the same clinical criteria. The clinical diagnosis would then serve as the standard to determine the accuracy of HCMV-miR-US4-1 as a biomarker for the prediction of efficacy of IFNα therapy. As shown in Fig. 4, serum levels of HCMV-miR-US4-1 did confirm the ability of HCMV-miR-US4-1 to be a potential biomarker for predicting the outcome of CHB patients who received IFNα therapy. Patients whose serum levels of HCMV-miR-US4-1 were above the cutoff point generally responded poorly to IFNα therapy, whereas patients whose serum levels of HCMV-miR-US4-1 were below the cutoff point responded effectively to IFNα therapy. Specifically, as a biomarker, serum levels of HCMV-miR-US4-1 correctly predicted 42 out of 50 CHB patients who were responsive to IFNα therapy (accuracy rate = 84.00%) and 33 out of 46 CHB patients who were non-responsive to IFNα therapy (accuracy rate = 71.74%), respectively. For CHB patients who were responsive to IFNα therapy, the false positive and false negative rate of HCMV-miR-US4-1 as the biomarker were 26.00% and 16.00%, respectively. For CHB patients who were not responsive to IFNα therapy, the false positive and false negative rate of HCMV-miR-US4-1 as the biomarker were 17.39% and 28.25%, respectively.

## Discussion

Recent studies by us and others have shown that circulating endogenous miRNAs carrying disease-specific information can act as non-invasive or minimally invasive biomarkers for various dysfunctions^19^,^20^,^21^,^22^,^23^,^24^. In this study, we demonstrated that serum levels of HCMV-encoded miRNAs, particularly HCMV-miR-US4-1, could accurately predict the outcomes of CHB patients following IFNα therapy.

Screening the serum levels of 13 HCMV-encoded miRNAs by qRT-PCR, using serum samples from CHB patients arranged in training and testing sets, clearly showed the differential expression pattern of HCMV-miR-US4-1 and HCMV-miR-HL148D in CHB patients who were responsive or non-responsive to IFNα therapy. As shown in Fig. 3, using HCMV-miR-US4-1 or HCMV-miR-HL148D as a biomarker, we can clearly separate the CHB patients into IFNα responsive and non-responsive groups. More importantly, through the perspective study employing 96 new CHB patients, we found that HCMV-miR-US4-1 alone had the power to predict the efficacy of IFNα therapy for individual CHB patients. The accuracy of the serum HCMV-miR-US4-1-based biomarker for predicting the outcome of IFNα therapy is over 80% (Fig. 4). In other words, based on the serum levels of HCMV-miR-US4-1 in individual CHB patients, we have considerable confidence to make a correct prediction whether the CHB patient is suitable for IFNα therapy or not. Like endogenous circulating miRNAs, which are well-protected from RNases and remain stable even after being subjected to harsh conditions, HCMV-encoded miRNAs are also highly stable in human serum and readily detected by qRT-PCR. Therefore, determination of HCMV-encoded miRNA signatures in patient serum, using qRT-PCR, is a clinically applicable procedure. Due to the simplicity and reproducibility of obtaining a blood sample, easily testable HCMV miRNA-based biomarkers found in blood serum may have great potential for the prediction of patient outcomes following specific therapy procedures.

HCMV is a β-herpesvirus that is ubiquitously present in the human population and persists for the lifetime of the host^16^. Infection with HCMV is usually subclinical in healthy adults, but can cause serious disease in populations with compromised immune systems, such as HIV-infected people or organ transplant recipients^25^. HCMV is also the leading viral cause of congenital birth defects in the developed world^26^. Given that liver cells, including hepatocytes, bile duct epithelial cells and stromal cells, can be infected by various HCMV strains^27^, it is likely that HCMV infection of liver cells would affect their response to anti-viral drug treatment. It is interesting that we found that only two HCMV-encoded miRNAs, HCMV-miR-US4-1 and HCMV-miR-UL148D, but no other HCMV miRNAs, were differentially expressed in the serum samples of CHB patients who were IFNα non-responsive compared to those patients who were IFNα responsive. This result, however, may suggest that HCMV encodes different miRNAs at different stage of infection. Indeed, various expression profiles of HCMV miRNAs have been reported during active virus infection or HCMV latency^28^.

Interferon signaling and its stimulated genes plays a fundamental role in host anti-viral responses^29^. Identification of the association between the specific circulating HCMV-encoded miRNAs and the efficacy of IFNα therapy for individual CHB patients may also provide evidence that HCMV infection can influence the host immune response of CHB patients to IFNα therapy. Although the underlying mechanistic basis is still unclear, it has been suggested that HCMV-encoded miRNAs play a critical role in modulating host immune surveillance, cell-cycle control and tumor progression^28^,^30^,^31^. MiR-UL112-1 was found to target a cellular gene that encodes the major histocompatibility complex (MHC) class I–related chain B, allowing viral escape from the natural killer cell–mediated immune response^30^. Another HCMV-encoded miRNA, miR-US25-1, was reported to downregulate many host genes associated with cell-cycle control and tumor progression by interacting with the 5′ UTR of a target mRNA^28^. In particular, Kim *et al.*^31^ showed that HCMV-miR-US4-1 could specifically downregulate aminopeptidase ERAP1 expression during viral infection, leading to a decrease in the MHC class I–presented peptides on the cell surface of CD8^+^ T cells, a critical step for the killing of virus-infected or transformed cells. Because CD8^+^ T cell-mediated killing of virus-infected cells is also a major mechanism of IFNα therapy in CHB patients, identification of higher levels of serum HCMV-miR-US4-1 in CHB patients who were non-responsive to IFNα therapy, compared with CHB patients who were responsive to IFNα therapy, may suggest that inhibition of MHC class I–presented HBV-derived peptides on the HBV virus-infected cell surface to CD8^+^ T cells by HCMV-miR-US4-1 likely contributes to the ineffective response of CHB patients to IFNα treatment. According to this hypothesis, the ERAP1-mediated trimming of HBV-derived peptides was inhibited by HCMV-miR-US4-1, which led to infected cells being less susceptible to HBV-specific cytotoxic T lymphocytes (CTLs) during IFNα therapy.

This is the first study to report a link between HCMV miRNAs and the outcome of IFNα treatment in CHB patients. However, the present study has some limitations. Our findings from Chinese CHB patients may not be generalizable to other populations because HCMV infection rates may vary across populations. In addition, the response to HCMV infection may be different among different ethnic groups. A full understanding of the target genes of HCMV-miR-US4-1 and the molecular mechanisms by which the serum level of this HCMV-encoded miRNA is linked to the effect of IFNα on CHB patients may lead to a broad clinical application. Therefore, the generalization of the serum HCMV-encoded miRNA signatures identified in this study warrants additional studies in various ethnic populations, preferably in prospective studies for IFNα therapy response evaluations of CHB patients.

## Methods

### Patient characteristics and clinical features

This study protocol conformed to the ethical guidelines of the 1975 Declaration of Helsinki and was approved by the Institutional Review Board of Nanjing University and Nanjing Medical University, Nanjing, China. A signed informed consent was obtained from each participant. A total of 152 CHB patients received IFNα therapy at the Nanjing Second Hospital (Nanjing, China) between 2012 and 2013. As shown in Table 1, all patients were hepatitis B virus surface antigen (HBsAg) positive. The levels of alanine aminotransferase (ALT) and aspartate aminotransferase (AST) of all CHB patients were at least two-fold higher than the upper limits of normal. The levels of HBV-DNA were all above 1000 copies/ml. Routine blood tests, such as white blood cell count, red blood cell count and platelet count, were all within the normal range.

### Patient serum collection, INFα treatment and classification

As depicted in Fig. 1, 5 ml of venous blood was drawn from individual participants prior to his/her INFα therapy. To harvest cell-free serum, the blood was drawn into a sterile tube without anticoagulant. After leaving the tube upright for 20 minutes, samples were centrifuged at 1500 *g* for 10 minutes at 20 °C and the supernatant serum was immediately removed and stored at −80 °C until analysis. For IFNα therapy, CHB patients were injected daily or every two days with recombinant human IFNα (6 × 10^6^ U) for a course of three months. Titers of HBV-DNA in individual patients were monitored at least twice during the course of therapy. At the end of the course of INFα therapy, patients were classified in INFα responsive (INFα-RES) and non-responsive (INFα-NRS) groups based on a clinical diagnosis. Specifically, for the INFα-RES group, the levels of HBV-DNA after treatment were less than 10% of the HBV-DNA levels before IFNα therapy, with normal AST and ALT levels. For the INFα-NRS group, the serum levels of HBV-DNA were greater than 10% of the HBV-DNA levels before IFNα therapy, with high levels of AST and ALT. To screen HCMV miRNAs in patient serum samples for biomarkers indicating the possible efficacy of INFα treatment, we randomly assigned serum samples from 56 CHB patients into a training set (20 samples) and a validation set (36 samples). To test the accuracy of the identified biomarker, 96 new CHB patients were recruited for a perspective study. In this study, blood samples were drawn from each CHB patient prior to the INFα therapy, and the efficacy of INFα therapy for these individual CHB patients was immediately determined using the identified HCMV miRNA-based biomarker. After the course of therapy, patients were subjected to clinical examinations. The accuracy of HCMV miRNA-based biomarker in predicting the outcome of INFα treatment is obtained by comparing the efficacy of the INFαpredicted by the HCMV miRNA-based biomarker with that determined by clinical diagnosis.

### RNA isolation and qRT-PCR assay

Total RNA was extracted from serum using the miRNeasy Mini Kit (Qiagen, Hilden, Germany) according to the manufacturer’s instructions. At each step, from serum purification to qRT-PCR, equal volumes of serum samples were processed. The qRT-PCR assays were performed in triplicate using an ABI 7500 instrument (Applied Biosystems, Foster City, CA). Expression levels of miRNAs were calculated using the C_T_ values. The ratio of the 2 groups of serum miRNAs were calculated by using the equation 2^−△G^, in which ΔG = C_T group1_ − C_T group2_. All primers used are available upon request.

### Enzyme-linked immunosorbent assay (ELISA)

ELISA was performed to detect anti-HCMV IgG and IgM antibodies in plasma using an HCMV IgG/IgM kit (DIA PRO Diagnostic Bioprobes, Milano, Italy) according to the manufacturer’s instructions. For the IgG-ELISA, an ELISA value of <0.5 IU/ml was considered a negative result, and a value of >0.5 IU/ml was considered a positive result, indicating prior exposure to HCMV. For the IgM-ELISA, the test results were calculated using the optical density (OD) value at 450 nm, and the cut-off value for positivity was OD > 1.2, indicating acute infection with HCMV. A value < 1.0 was considered a negative result and a value between 1.0 and 1.2 was considered equivocal.

### Quantitative PCR

To investigate the association between INFα-RES group and INFα-NRS group, we tested the seropositivity and copy number of HCMV by quantitative PCR in 28 IFNα responsive patients and 28 IFNα non-responsive patients of training and testing sets. We extracted HCMV DNA from 200 μL separated plasma using the QIAamp DNA Mini kit (Qiagen) according to the manufacturer’s protocol. We amplified HCMV DNA by TaqMan real-time PCR with HCMV-specific primers according to previous publication^13^. The 2-step thermocycling procedure consisted of 45 cycles of denaturation at 95 °C for 15 seconds and annealing and extension at 60 °C for 60 seconds. The PCR product was hybridized to an HCMV-specific, FAM-labeled probe. Serving as a positive control, plasmid DNA containing the HCMV target sequence was used in separate reactions on each TaqMan assay plate. Results were expressed as copies per 1 mL plasma.

### Statistical analysis

Clinical characteristics between the two groups were statistically analyzed using a Student’s *t*-test and χ^2^ test (Table 1). Data from the qRT-PCR assays were also statistically analyzed using a Student’s *t*-test. The results were presented as the mean ± SEM. Values of *P* < 0.05 were considered statistically significant. The receiver operating characteristic (ROC) curves were used to evaluate the diagnostic effects of the identified HCMV miRNA-based biomarkers. All statistical analyses were performed using GraphPad Prism 5 software.

## Additional Information

**How to cite this article**: Pan, Y. *et al.* Circulating human cytomegalovirus-encoded HCMV-miR-US4-1 as an indicator for predicting the efficacy of IFNα treatment in chronic hepatitis B patients. *Sci. Rep.* **6**, 23007; doi: 10.1038/srep23007 (2016).

## Supplementary Material

Supplementary Information

## Figures and Tables

**Table 1:** CHB patient characteristics (including training set, validation set and perspective study group).

Characteristics		IFNα-responsive (n = 74)	IFNα-non-responsive (n = 78)	p-value
Age (years)		32.27 ± 1.07	29.5 ± 0.94	0.059^a^
HBV-DNA(10^7^ copies/ml)		4.85 ± 1.67	6.15 ± 1.52	0.562^a^
n = 152		n = 74	n = 78	
Sex	Male	58	53	0.123^b^
	Female	16	25	
Adjuvant therapy	Yes	13	7	0.096^b^
	No	61	71	
Leucocytes (10^9^/L)		4.55 ± 0.19	4.83 ± 0.16	0.262^a^
Platelets(10^9^/L)		126.58 ± 5.49	142.15 ± 5.43	0.484^a^
Total bilirubin (μmol/L)		15.29 ± 1.36	12.67 ± 0.71	0.091^a^
Albumin (g/L)		46.39 ± 1.12	44.89 ± 0.44	0.239^a^
Globulin (g/L)		27.04 ± 0.50	27.28 ± 0.62	0.812^a^
ALT (U/L)		161.51 ± 25.63	177.62 ± 18.35	0.619^a^
AST (U/L)		88.25 ± 11.21	93.16 ± 7.85	0.751^a^
Anti-HBc (n = 101)		9.73 ± 0.29 (n = 57)	9.72 ± 0.34 (n = 44)	0.999^a^
HBsAg	Positive	72	67	
	Negative	0	0	
GTP (U/L)		82.95 ± 7.93	63.82 ± 5.90	0.064^a^
ALP (U/L)		82.83 ± 5.58	76.45 ± 3.53	0.332^a^
α-fetoprotein (ng/ml) (n = 63)		19.82 ± 8.37 (n = 33)	18.84 ± 8.07 (n = 30)	0.936^a^
Response after treatment	HBV-DNA < 500 copies/ml	57	0	
	HBV-DNA after IFNα therapy/before IFNα therapy < 0.1	74	0	
	HBeAg negative	20	0	
	HBsAg negative	20	0	

^a^student-t test.

^b^two-sided χ2 test. ALT: alanine aminotransferase; AST: aspartate aminotransferase; GTP: glutamyltranspeptidase; ALP: alkaline phosphatase. HBV-DNA < 500 copy/ml or HBV-DNA level after IFNα therapy/HBV-DNA level before IFNα therapy <0.1 was considered as IFNα effective.
