# Supplementary material for: Circulating human cytomegalovirus-encoded HCMV-miR-US4-1 as an indicator for predicting the efficacy of IFNα treatment in chronic hepatitis B patients
Source: Sci Rep. 2016 Mar 10;6:23007. doi: 10.1038/srep23007 (PMC4785337; doi:10.1038/srep23007)
Supplement: Supplementary Information [file srep23007-s1.doc]

**Circulating human cytomegalovirus-encoded HCMV-miR-US4-1 as an indicator for predicting the efficacy of IFNα treatment in chronic hepatitis B patients**

Yi Pan^1,2+^, Nan Wang^1,2+^, Zhenxian Zhou^3+^, Hongwei Liang^1,2^, Chaoyun Pan^1,2^, Dihan Zhu^1,2^, Fenyong Liu^4^, Yujing Zhang^1,2*^, Chen-Yu Zhang^1,2*^, and Ke Zen^1,2*^

^1^State Key Laboratory of Pharmaceutical Biotechnology, Nanjing Advanced Institute for Life Sciences (NAILS), Nanjing University, 22 Hankou Road, Nanjing, Jiangsu 210093, China. ^2^Jiangsu Engineering Research Center for microRNA Biology and Biotechnology, School of Life Sciences, Nanjing University, Nanjing 210093, China; ^3^Clinical Laboratory, Nanjing Second Hospital, Nanjing 210003, China; ^4^Department of Virology, University of California School of Public Health, Berkeley, CA 94720, USA.

**Running title: serum HCMV-miR-US4-1 as biomarker for IFNα efficacy**

^+^These authors contributed equally to this work

^*^To whom correspondence may be addressed:

Ke Zen, PhD, Chen-Yu Zhang, M.D, PhD and/or Yujing Zhang, PhD

State Key Laboratory of Pharmaceutical Biotechnology

NAILS, Nanjing University

22 Hankou Road 22

Nanjing, Jiangsu 210093, China

E-mail: [kzen@nju.edu.cn](mailto:kzen@nju.edu.cn), [cyzhang@nju.edu.cn](mailto:cyzhang@nju.edu.cn) and/or

**Supplementary Table S1. The detailed information of the patients in INFα-RES group.**

|  | | | **Before treatment** | | | | | | | | | | | | | **After treatment** | | |
| --- | --- | --- | --- | --- | --- | --- | --- | --- | --- | --- | --- | --- | --- | --- | --- | --- | --- | --- |
| **Num** | **Sex** | **Age** | **HBV**  **-DNA** | **ADJ THER** | **ALT**  **(U/L)** | **AST**  **(U/L)** | **GTP**  **(U/L)** | **ALP**  **(U/L)** | **ALB**  **(g/L)** | **GLU**  **(g/L)** | **HBsAg** | **Anti-**  **HBc** | **WBC**  **(10^9^/L)** | **PLT**  **(10^9^/L)** | **AFP**  **(ng/ml)** | **HBV -DNA**  **<500** | **HBeAg** | **HBsAg** |
| 1 | M | 21 | 4.10E+05 | No | 169.2 | 36 | 62.2 | 79.6 | 43.6 | 23.2 | + | 9.37 | 6.14 | 188 | UKN | c | UKN | + |
| 2 | M | 31 | 8.70E+08 | Yes | 75 | 59.9 | 80 | 85.8 | 44.2 | 28.5 | + | 12.26 | 3.33 | 60 | UKN | Yes | - | UKN |
| 3 | M | 28 | 3.20E+06 | No | 55.2 | 21.1 | 55.5 | 47.5 | 48.1 | 24.5 | + | 8.36 | 6.57 | 143 | 2.57 | Yes | - | + |
| 4 | M | 34 | 6.60E+08 | No | 54 | 50.9 | 86.8 | 95.4 | 50.8 | 29.7 | + | 10.15 | 3.92 | 54 | UKN | Yes | + | + |
| 5 | M | 29 | 5.60E+05 | No | 428.2 | 202.2 | 167 | 115.3 | 115.3 | 30.7 | + | 8.49 | 4.06 | 244 | 34.68 | Yes | + | + |
| 6 | M | 34 | 4.20E+07 | No | 40.4 | 42 | 29 | 62.2 | 47 | 28.4 | + | 10.16 | 3.99 | 136 | 5.38 | Yes | + | - |
| 7 | M | 37 | 8.80E+06 | No | 192.3 | 192.3 | 137.8 | 100.2 | 44.3 | 29.7 | + | 13.01 | 3.39 | 118 | UKN | Yes | + | - |
| 8 | M | 34 | 1.20E+05 | Yes | 16.9 | 19.7 | 15.6 | 71.3 | 53 | 25 | + | 11.22 | 2.96 | 135 | 3.04 | Yes | - | + |
| 9 | M | 42 | 3.20E+08 | No | 240 | 153.5 | 196 | 87.3 | 35.3 | 33.5 | + | 8.36 | 3.46 | 66 | UKN | Yes | + | - |
| 10 | M | 37 | 2.30E+06 | No | 295.2 | 66.3 | 57 | 68.3 | 47.6 | 19.6 | + | 6.87 | 3.96 | 153 | UKN | Yes | + | + |
| 11 | M | 14 | 1.10E+08 | No | 188.8 | 259.1 | 131.4 | 383.7 | 45.1 | 29.4 | + | 7.78 | 4.07 | 194 | UKN | Yes | UKN | UKN |
| 12 | M | 20 | 5.90E+06 | No | 1358 | 426.5 | 94.8 | 93.1 | 42.8 | 32.1 | + | 9.98 | 4.92 | 139 | 36.72 | Yes | - | + |
| 13 | F | 33 | 4.30E+06 | No | 26 | 24.3 | 19 | 36.1 | 52.7 | 27.1 | + | 11.51 | 3.68 | 182 | 1.21 | Yes | - | + |
| 14 | M | 28 | 4.00E+04 | No | 124.7 | 89.5 | 69.4 | 74.5 | 50.8 | 30.4 | + | 11.91 | 6.1 | 94 | UKN | Yes | UKN | UKN |
| 15 | M | 44 | 4.40E+06 | No | 91.5 | 48.7 | 316.2 | 106.9 | 42.6 | 40.4 | + | UKN | 5.18 | 120 | 25.12 | Yes | + | + |
| 16 | M | 34 | 6.20E+06 | Yes | 38.8 | 21.3 | 24.2 | 67.7 | 47.5 | 34.3 | + | 6.13 | 8.94 | 218 | UKN | Yes | + | + |
| 17 | M | 50 | 1.10E+04 | No | 184.9 | 40.7 | 247.2 | 87.9 | 45.4 | 35.8 | + | 7.69 | 4.01 | 128 | 49.07 | Yes | + | + |
| 18 | F | 25 | 3.30E+06 | Yes | 184.4 | 68.6 | 45.1 | 60.5 | 49.1 | 27.4 | + | 8.75 | 4.22 | 63 | UKN | Yes | - | + |
| 19 | M | 39 | 2.50E+07 | No | 1251 | 574.9 | 236.7 | 111.5 | 41.3 | 22 | + | 10.22 | 5.6 | 146 | UKN | Yes | UKN | UKN |
| 20 | M | 43 | 4.60E+07 | No | 53.8 | 33 | 43.7 | 60.2 | 43.9 | 30 | + | UKN | 2.48 | 60 | 1.79 | Yes | UKN | UKN |
| 21 | F | 38 | 3.10E+07 | No | 167.2 | 95.8 | 31.3 | 64.9 | 47.5 | 32.5 | + | UKN | 4.13 | 208 | 1.86 | Yes | UKN | UKN |
| 22 | F | 25 | 2.20E+07 | No | 23.1 | 28.9 | 12.2 | 41.4 | 51.3 | 28.7 | + | UKN | 3.14 | 82 | UKN | Yes | + | + |
| 23 | F | 29 | 4.10E+07 | No | 14.6 | 20.8 | 16 | 45.2 | 43.6 | 36.9 | + | UKN | 3.96 | 112 | UKN | Yes | UKN | UKN |
| 24 | M | 38 | 4.10E+07 | No | 94.2 | 82.5 | 94.6 | 71.7 | 50.3 | 28.8 | + | 15.89 | 4.58 | 197 | 3.15 | Yes | UKN | UKN |
| 25 | M | 30 | 5.20E+06 | No | 56 | 31.8 | 114.4 | 106.8 | 46.9 | 28 | + | 1.3 | 4.7 | 142 | UKN | Yes | + | + |
| 26 | M | 31 | 7.10E+06 | No | 38.2 | 30 | 22.5 | 67 | 49.8 | 24.8 | + | UKN | 4.36 | 137 | 4.19 | Yes | UKN | + |
| 27 | M | 36 | 1.10E+06 | No | 69.5 | 53.6 | 76.7 | 54.5 | 47.5 | 27.2 | + | 6.93 | 3.2 | 86 | 9.47 | Yes | + | + |
| 28 | M | 37 | 1.00E+07 | No | 88.9 | 46.7 | 65.9 | 52.4 | 42.3 | 25.9 | + | 11.09 | 8.78 | 173 | UKN | Yes | - | + |
| 29 | F | 23 | 3.50E+06 | No | 521.9 | 263.8 | 66 | 70.7 | 46.4 | 28.4 | + | 10.2 | 6.68 | 280 | 7.27 | Yes | UKN | UKN |
| 30 | F | 48 | 9.00E+05 | No | 26.3 | 31.3 | 42.6 | 74.7 | 48 | 31 | + | UKN | 2.72 | 123 | 2.81 | Yes | + | + |
| 31 | M | 47 | 1.40E+07 | Yes | 18.6 | 19 | 0 | 42.6 | 50 | 25.3 | + | 9.25 | 3.39 | 166 | 3.39 | Yes | + | + |
| 32 | M | 32 | 2.10E+07 | No | 95.4 | 56.9 | 95.6 | 60.8 | 42.9 | 22.3 | + | 7.66 | 3.87 | 82 | UKN | Yes | UKN | - |
| 33 | F | 41 | 1.50E+07 | No | 78.3 | 56.2 | 30.2 | 17.4 | 43.5 | 24.6 | + | 9.81 | 4.01 | 69 | UKN | Yes | UKN | UKN |
| 34 | M | 29 | 3.10E+06 | No | 50.5 | 24.9 | 74.6 | 45.5 | 49.9 | 28 | + | 6.18 | 7.95 | 197 | UKN | Yes | + | + |
| 35 | M | 47 | 2.30E+07 | No | 38.1 | 30.5 | 52.9 | 82.4 | 43.5 | 21.4 | + | 11.41 | 2.45 | 103 | 4.44 | Yes | UKN | UKN |
| 36 | F | 43 | 1.40E+07 | No | 74.8 | 74.1 | 36.6 | 59.1 | 49 | 30.2 | + | UKN | 4.01 | 55 | UKN | Yes | UKN | - |
| 37 | M | 23 | 3.20E+05 | Yes | 24.5 | 15.6 | 17 | 74.3 | 15.6 | 26.9 | + | 8.74 | 3.95 | 121 | UKN | Yes | - | - |
| 38 | M | 29 | 5.50E+05 | Yes | 129.8 | 73.1 | 77.2 | 46.4 | 50.9 | 22.1 | + | 11.67 | 4.19 | 73 | UKN | Yes | - | - |
| 39 | M | 54 | 7.60E+05 | No | 24.7 | 23 | 18.3 | 100.6 | 51 | 23.1 | + | 14.74 | 9.7 | 130 | 2.05 | Yes | - | - |
| 40 | M | 25 | 3.20E+07 | Yes | 73.7 | 32.8 | 45 | 54.8 | 51.3 | 21.3 | + | 12.62 | 5.24 | 131 | 2.53 | Yes | + | + |
| 41 | M | 28 | 3.20E+07 | No | 75.6 | 51.8 | 140.7 | 79.1 | 46.8 | 27.7 | + | 10.15 | 5.41 | 144 | UKN | Yes | - | + |
| 42 | M | 51 | 1.80E+06 | Yes | 44.1 | 39.5 | 38.5 | 115.7 | 46.6 | 24.4 | + | 13.4 | 7.3 | 102 | 3.83 | Yes | + | - |
| 43 | F | 27 | 2.80E+06 | No | 40.7 | 35.6 | 14.1 | 53.2 | 48.7 | 38.3 | + | 9.2 | 3.4 | 145 | UKN | Yes | + | - |
| 44 | F | 30 | 7.90E+06 | No | 26.4 | 25.7 | 15.4 | 61.1 | 49 | 28.7 | + | 11.12 | 2.04 | 84 | UKN | Yes | + | - |
| 45 | M | 41 | 2.20E+06 | No | 37.4 | 29.9 | 33.8 | 54.7 | 54.7 | 27.4 | + | UKN | 3.52 | 84 | UKN | Yes | - | UKN |
| 46 | M | 27 | 6.70E+05 | No | 53.4 | 37.7 | 219.3 | 219.3 | 44.6 | 24.3 | + | 6.66 | 3.71 | 152 | 9.68 | Yes | - | - |
| 47 | M | 38 | 4.10E+06 | Yes | 277.8 | 124.8 | 35.7 | 62.6 | 46.3 | 27.7 | + | 1.23 | 3.75 | 130 | UKN | No | + | + |
| 48 | M | 27 | 4.10E+07 | No | 123.7 | 52 | 102 | 67.8 | 44.8 | 22.1 | + | 11.93 | 8.27 | 150 | UKN | No | + | + |
| 49 | F | 32 | 2.20E+07 | Yes | 34 | 25.8 | 16.3 | 70.6 | 52.4 | 26.6 | + | 10.61 | 4.87 | 146 | UKN | No | + | + |
| 50 | M | 41 | 1.30E+05 | No | 56.7 | 74.3 | 35.2 | 53.5 | 49.1 | 20.2 | + | 13.89 | 4.71 | 115 | 1.75 | Yes | + | + |
| 51 | M | 21 | 5.40E+08 | No | 575.1 | 368.5 | 138.6 | 73.1 | 44.8 | 25 | + | 8.27 | 5.63 | 139 | UKN | No | + | + |
| 52 | F | 21 | 2.10E+07 | No | 216.3 | 128.3 | 109.3 | 92 | 36.5 | 26 | + | 8.37 | 6.3 | 92 | UKN | No | + | + |
| 53 | M | 24 | 2.80E+06 | No | 58.6 | 71.4 | 121.5 | 92.5 | 40.7 | 25 | + | 15.71 | 3.01 | 91 | 284.2 | No | + | + |
| 54 | M | 21 | 1.20E+07 | No | 138.3 | 84.8 | 306.4 | 149 | 43.9 | 23.2 | + | 15.55 | 5.57 | 163 | 7.78 | No | - | + |
| 55 | M | 35 | 4.90E+05 | No | 34 | 24.2 | 74.9 | 68.8 | 43.6 | 23.2 | + | 13.72 | 2.38 | 102 | 9.2 | Yes | - | - |
| 56 | M | 52 | 3.20E+05 | No | 124.7 | 41 | 19 | 51.1 | 42.9 | 22.1 | + | 10.12 | 4.01 | 149 | 2.45 | Yes | + | + |
| 57 | F | 27 | 2.00E+08 | Yes | 187 | 139.6 | 47.7 | 68.4 | 43 | 29.4 | + | 13.24 | 6.07 | 129 | UKN | Yes | + | + |
| 58 | M | 31 | 1.20E+07 | No | 195.2 | 81.9 | 143.1 | 106.1 | 40.7 | 28.9 | + | 10.19 | 4.2 | 245 | 3.68 | Yes | + | + |
| 59 | M | 41 | 9.00E+04 | No | 297.3 | 187.1 | 64.8 | 73.3 | 39.8 | 25.1 | + | UKN | 6.28 | 124 | UKN | No | + | + |
| 60 | M | 41 | 5.50E+04 | No | 253.7 | 192.2 | 30.2 | 56.4 | 41.4 | 20.8 | UKN | UKN | 2.99 | 102 | 9.2 | Yes | + | + |
| 61 | M | 24 | 6.10E+06 | Yes | 48.7 | 31.1 | 31.1 | 86.9 | 48.8 | 26.9 | UKN | UKN | 4.08 | 67 | UKN | No | + | UKN |
| 62 | M | 22 | 1.10E+08 | No | 310.3 | 84.4 | 92.4 | 118.6 | 47.7 | 25.4 | + | 12.32 | 4.59 | 131 | UKN | No | + | UKN |
| 63 | M | 36 | 7.30E+06 | No | 117.9 | 53.2 | 78.3 | 61.1 | 41.2 | 30.6 | + | 12.18 | 3.1 | 86 | 21 | No | - | + |
| 64 | M | 30 | 5.00E+05 | No | 187.8 | 85.6 | 139.1 | 96.2 | 53.7 | 26.2 | + | 14.11 | 4.5 | 148 | UKN | No | + | + |
| 65 | F | 33 | 2.90E+04 | No | 63.6 | 44.9 | 28.5 | 36.8 | 41.3 | 24.7 | + | UKN | 4.99 | 93 | UKN | Yes | + | + |
| 66 | M | 40 | 1.10E+08 | No | 332.1 | 216.7 | 151.4 | 99 | 35 | 29.6 | + | UKN | 2.6 | 50 | UKN | Yes | - | + |
| 67 | F | 20 | 5.50E+05 | No | 122.3 | 61.8 | 21.8 | 47.4 | 45.2 | 26.3 | + | UKN | 4.11 | 160 | UKN | Yes | + | + |
| 68 | M | 16 | 1.80E+06 | No | 352.7 | 118 | 96.7 | 218.4 | 45.5 | 17.3 | + | 12.58 | 2.12 | 73 | UKN | No | - | + |
| 69 | M | 25 | 8.30E+05 | No | 94.2 | 37.5 | 54.8 | 79.5 | 41 | 31.5 | + | UKN | 5.06 | 106 | 4 | Yes | UKN | UKN |
| 70 | M | 40 | 6.50E+04 | No | 50.7 | 20.6 | 231.3 | 85.4 | 49.3 | 27.3 | + | UKN | 6.06 | 157 | 6.26 | Yes | + | - |
| 71 | M | 29 | 1.50E+07 | No | 151.2 | 228.6 | 152.6 | 110.7 | 40.4 | 23.6 | + | 12.76 | 2.61 | 94 | 10.8 | No | + | UKN |
| 72 | M | 29 | 3.10E+07 | No | 290.1 | 102.9 | 91.1 | 77.9 | 39.9 | 32.7 | + | 9.34 | 3.59 | 112 | UKN | No | - | + |
| 73 | M | 4 | 1.10E+05 | No | 59.1 | 56.8 | 38.7 | 139 | 40.2 | 19.8 | + | 7.61 | 3.39 | 62 | 79.4 | No | UKN | UKN |
| 74 | M | 22 | 4.60E+04 | No | 144.7 | 50.6 | 119.7 | 76 | 51.5 | 24 | + | 8.8 | 6.8 | 131 | UKN | No | - | - |

Num: patients number; ADJ THER: adjuvant therapy; ALT: alanine aminotransferase; AST: aspartate aminotransferase; GTP: glutamyl transpeptidase; ALP: alkaline phosphatase; ALB: albumin; GLU: globulin; WBC: leucocytes; PLT: platelets; AFP: α-fetoprotein. F: female; M: male; +: positive; -: negative; UKN: unknown. HBV-DNA unit: copy/ml.

**Supplementary Table S2.** The detailed information of the patients in INFα-NRS group.

|  | | | **Before treatment** | | | | | | | | | | | | | **After treatment** | | |
| --- | --- | --- | --- | --- | --- | --- | --- | --- | --- | --- | --- | --- | --- | --- | --- | --- | --- | --- |
| **Num** | **Sex** | **Age** | **HBV**  **-DNA** | **ADJ THER** | **ALT**  **(U/L)** | **AST**  **(U/L)** | **GTP**  **(U/L)** | **ALP**  **(U/L)** | **ALB**  **(g/L)** | **GLU**  **(g/L)** | **HBsAg** | **Anti-**  **HBc** | **WBC**  **(10^9^/L)** | **PLT**  **(10^9^/L)** | **AFP**  **(ng/ml)** | **HBV -DNA**  **<500** | **HBeAg** | **HBsAg** |
| 1 | F | 22 | 3.70E+08 | Yes | 90.8 | 41.2 | 29.6 | 73.9 | 39 | 25.3 | + | 14.89 | 3.56 | 149 | UKN | No | + | UKN |
| 2 | M | 29 | 1.00E+06 | No | 308.2 | 81.3 | 110.8 | 38.4 | 44.9 | 21.9 | + | 14.03 | 2.61 | 110 | 72.23 | No | UKN | UKN |
| 3 | M | 29 | 4.10E+04 | No | 99.5 | 73.9 | 24.4 | 71.6 | 41.9 | 27.5 | UKN | UKN | 6.3 | 152 | UKN | No | UKN | UKN |
| 4 | M | 32 | 1.80E+04 | Yes | 63.7 | 39.1 | 25.6 | 61.4 | 47.6 | 20.4 | + | 14.54 | 5.8 | 119 | 2.54 | No | UKN | UKN |
| 5 | F | 56 | 9.00E+05 | No | 126.4 | 116.1 | 108.6 | 100.1 | 40.2 | 35.7 | UKN | UKN | 6.05 | 88 | 6.43 | No | UKN | UKN |
| 6 | M | 42 | 2.80E+08 | No | 179.6 | 123.6 | 226.8 | 97.1 | 34.9 | 29.5 | + | 6.66 | 4.26 | 112 | UKN | No | UKN | UKN |
| 7 | F | 22 | 3.20E+07 | No | 100.4 | 65.8 | 11.2 | 78.5 | 51.5 | 37 | + | 11.78 | 5.55 | 182 | 2.88 | No | + | + |
| 8 | F | 30 | 9.20E+03 | No | 179.1 | 110.2 | 37.7 | 74.2 | 49.2 | 26.8 | + | 6.96 | 2.64 | 148 | 2.74 | No | + | + |
| 9 | M | 22 | 1.70E+06 | No | 202.6 | 84.8 | 89 | 100.3 | 44.2 | 21.4 | + | 9.39 | 3.91 | 132 | UKN | No | + | + |
| 10 | M | 34 | 5.70E+05 | No | 179.9 | 92.3 | 78.8 | 48 | 45.2 | 25.2 | + | 6.76 | 7.22 | 146 | UKN | No | + | + |
| 11 | M | 28 | 5.00E+03 | No | 167.9 | 117 | 135 | 78.8 | 43.4 | 23.3 | UKN | UKN | 4.7 | 200 | UKN | No | UKN | UKN |
| 12 | M | 11 | 1.00E+08 | No | 362.8 | 176.2 | 41 | 214.2 | 44.4 | 24.7 | + | 7.9 | 5.95 | 131 | UKN | No | + | + |
| 13 | F | 24 | 2.90E+03 | No | 275.8 | 130.9 | 84.4 | 71.5 | 44.5 | 25.4 | + | 6.4 | 2.54 | 82 | 9.4 | No | + | + |
| 14 | M | 32 | 1.30E+06 | No | 471.6 | 241.9 | 82.5 | 57.8 | 40.4 | 29.3 | + | 7 | 5.98 | 150 | UKN | No | + | + |
| 15 | F | 27 | 1.10E+04 | No | 75.1 | 59.7 | 52.4 | 41.9 | 43.9 | 33.2 | UKN | UKN | 2.62 | 120 | UKN | No | UKN | UKN |
| 16 | F | 29 | 8.60E+04 | No | 142.7 | 127.5 | 73.2 | 62.2 | 49.2 | 31.7 | + | 10.28 | 5.74 | 132 | 11.62 | No | + | + |
| 17 | M | 24 | 5.50E+06 | No | 73.7 | 33.1 | 23.2 | 59.5 | 50.5 | 25.2 | + | UKN | 5.72 | 142 | UKN | No | + | + |
| 18 | M | 50 | 1.40E+07 | No | 59.4 | 54.8 | 14.4 | 167.5 | 47.4 | 20.7 | + | 10.25 | 6.57 | 255 | 1.27 | No | + | + |
| 19 | M | 23 | 1.70E+06 | No | 58.1 | 61.4 | 36.3 | 43.8 | 47.9 | 21.8 | + | 9.74 | 6.3 | 114 | UKN | No | + | + |
| 20 | M | 24 | 1.20E+04 | No | 39.4 | 20.3 | 21.2 | 79 | 44.3 | 22.3 | + | UKN | 5.11 | 102 | 3.56 | No | + | + |
| 21 | M | 28 | 4.40E+07 | No | 84.7 | 57.1 | 49.9 | 65.4 | 44 | 29 | + | 8.81 | 3.56 | 161 | 14.33 | No | + | + |
| 22 | F | 26 | 9.50E+07 | No | 19.8 | 21.4 | 22.4 | 72.6 | 51.8 | 21 | + | 9.71 | 5.79 | 133 | 2.04 | No | + | + |
| 23 | M | 28 | 1.60E+06 | No | 53.1 | 33.6 | 47.8 | 65.4 | 44.4 | 20.9 | + | 9.81 | 5.86 | 144 | UKN | No | + | + |
| 24 | M | 44 | 1.60E+08 | No | 184.1 | 101.8 | 30.7 | 151.8 | 47.1 | 22.7 | UKN | UKN | 5.99 | 198 | 1.4 | No | + | + |
| 25 | M | 32 | 2.80E+06 | No | 254.9 | 138.4 | 35 | 58.6 | 47.5 | 32.8 | UKN | UKN | 4.92 | 223 | UKN | No | + | + |
| 26 | M | 14 | 1.00E+08 | No | 52.3 | 33.2 | 22.5 | 138.8 | 45.3 | 23.1 | + | UKN | 5.7 | 175 | UKN | No | + | + |
| 27 | M | 48 | 6.60E+05 | No | 296.1 | 118.9 | 55.5 | 76.7 | 43.5 | 22.8 | + | 7.03 | 3.13 | 86 | UKN | No | + | + |
| 28 | M | 15 | 2.40E+08 | No | 156.8 | 85.9 | 35.1 | 149.2 | 43.1 | 23.6 | + | UKN | 4.9 | 111 | UKN | No | + | + |
| 29 | M | 37 | 4.10E+05 | No | 339.3 | 156.4 | 78.6 | 65 | 40.6 | 40.6 | + | 9.8 | 6.03 | 158 | UKN | No | + | + |
| 30 | F | 30 | 8.20E+07 | No | 286.3 | 204.9 | 76.2 | 93.9 | 42.7 | 30.3 | + | 9.46 | 2.36 | 83 | UKN | No | + | + |
| 31 | M | 38 | 3.80E+06 | No | 430.4 | 246.7 | 164.3 | 73 | 49.7 | 49.7 | + | 8.55 | 5.32 | 162 | UKN | No | + | + |
| 32 | M | 47 | 5.00E+02 | No | 79 | 49.6 | 25.8 | 97.2 | 50.4 | 27.5 | + | 10.74 | 4.91 | 149 | 3.88 | No | + | + |
| 33 | M | 45 | 2.10E+08 | Yes | 563.5 | 184.4 | 80.8 | 77.6 | 40.1 | 29.5 | + | 8.43 | 3.85 | 147 | 15.47 | No | + | + |
| 34 | M | 20 | 2.30E+07 | No | 294.4 | 109.7 | 45.7 | 67.4 | 42 | 20.5 | + | 10.06 | 5.62 | 190 | UKN | No | + | + |
| 35 | F | 23 | 5.00E+02 | No | 305.7 | 375.4 | 249.4 | 101.1 | 40.1 | 30.7 | + | 9.7 | 5.17 | 72 | UKN | No | + | + |
| 36 | M | 34 | 1.10E+08 | No | 412.5 | 74.1 | 190.5 | 73.8 | 42 | 25.6 | UKN | UKN | 6.1 | 190 | 222.65 | No | UKN | UKN |
| 37 | F | 20 | 8.00E+03 | No | 115.4 | 72.8 | 50.7 | 25.4 | 41.5 | 30.2 | UKN | UKN | 4.74 | 211 | UKN | No | UKN | UKN |
| 38 | M | 29 | 1.30E+03 | No | 107.3 | 71.9 | 19.4 | 60.9 | 43.1 | 24.7 | + | 12.39 | 3.1 | 74 | UKN | No | UKN | UKN |
| 39 | F | 34 | 2.50E+07 | No | 352.9 | 212.9 | 187.8 | 68.1 | 45.1 | 30.9 | + | 11.15 | 5.4 | 161 | 10.91 | No | UKN | UKN |
| 40 | M | 29 | 4.40E+06 | No | 88.3 | 50.7 | 63.8 | 81.3 | 38.8 | 28.1 | + | 6.45 | 4.88 | 159 | 8.55 | No | + | + |
| 41 | M | 32 | 1.50E+08 | No | 214.4 | 99.5 | 23.8 | 58.7 | 47.1 | 27.5 | UKN | UKN | 4.99 | 156 | 10.14 | No | + | UKN |
| 42 | M | 21 | 3.00E+07 | No | 208.9 | 112 | 164.7 | 77.3 | 47.7 | 24.4 | + | 11.67 | 4.44 | 146 | 3.1 | No | + | + |
| 43 | M | 22 | 1.50E+08 | Yes | 1022.4 | 268.9 | 121.3 | 102.7 | 51.3 | 28.9 | + | 9.92 | 3.75 | 150 | UKN | No | UKN | UKN |
| 44 | F | 29 | 5.40E+03 | Yes | 16.2 | 17.8 | 10.9 | 74.1 | 45.8 | 24.6 | + | 13.07 | 4.47 | 179 | UKN | No | + | + |
| 45 | M | 22 | 7.30E+07 | No | 358.6 | 209.6 | 51.8 | 88.4 | 41.5 | 27.2 | + | 8.91 | 4.15 | 126 | UKN | No | + | + |
| 46 | M | 26 | 1.60E+07 | No | 141.5 | 80.3 | 78.8 | 103.4 | 46.4 | 31.3 | + | 8.21 | 6.27 | 163 | UKN | No | + | + |
| 47 | M | 23 | 1.60E+08 | Yes | 438.2 | 136.4 | 43.6 | 83.4 | 46.9 | 20.2 | + | UKN | 4.38 | 155 | 7.7 | No | UKN | UKN |
| 48 | F | 31 | 5.40E+07 | No | 68.4 | 50.8 | 31.7 | 45.8 | 38.9 | 24.9 | UKN | UKN | 5.99 | 160 | UKN | No | + | UKN |
| 49 | M | 27 | 1.30E+06 | No | 143.5 | 64.1 | 113.3 | 65.6 | 47.4 | 29 | + | 10.21 | 3.1 | 116 | UKN | No | + | + |
| 50 | M | 39 | 7.10E+06 | No | 64.2 | 45.4 | 16.9 | 50.7 | 44 | 21.6 | + | UKN | 5 | 106 | UKN | No | + | + |
| 51 | F | 19 | 3.10E+07 | No | 115.6 | 51.8 | 29.5 | 58 | 45.1 | 19.6 | + | 15.8 | 4.62 | 62 | UKN | No | + | + |
| 52 | M | 38 | 5.20E+04 | Yes | 55.9 | 38.8 | 31.2 | 63.9 | 46 | 35.3 | + | 13 | 4.12 | 163 | 4.8 | No | + | + |
| 53 | M | 36 | 1.10E+08 | No | 474.4 | 240.4 | 134.6 | 148.4 | 42.7 | 25.8 | + | UKN | 5.14 | 135 | UKN | No | + | + |
| 54 | M | 31 | 9.80E+07 | No | 101.7 | 41.8 | 57.2 | 71 | 44.8 | 23.9 | + | 10.83 | 4.5 | 64 | UKN | No | + | + |
| 55 | M | 35 | 1.10E+08 | No | 65.3 | 27.7 | 14.4 | 53.8 | 41 | 39.1 | + | 8.26 | 3.55 | 200 | 1.9 | No | + | + |
| 56 | F | 30 | 1.20E+08 | No | 57.4 | 37.4 | 7.5 | 46 | 39.4 | 25.5 | + | 8.5 | 4.2 | 137 | 1.3 | No | + | + |
| 57 | M | 31 | 4.10E+05 | No | 376.4 | 171.8 | 62.7 | 101.3 | 51.6 | 33.7 | + | 15.54 | 5.37 | 317 | UKN | No | + | + |
| 58 | M | 27 | 1.60E+07 | No | 22.1 | 16.8 | 29.6 | 51.2 | 52.1 | 24.3 | + | UKN | 4.59 | 159 | 6 | No | + | + |
| 59 | F | 42 | 1.00E+07 | No | 125 | 142.7 | 166.5 | 85.8 | 40 | 26.8 | + | 5.2 | 2.58 | 75 | 8.6 | No | + | UKN |
| 60 | F | 23 | 4.10E+07 | No | 27 | 31.7 | 18.1 | 59.3 | 43.8 | 31.7 | + | UKN | 3.09 | 66 | UKN | No | + | + |
| 61 | F | 25 | 2.10E+07 | No | 125 | 50.7 | 27.8 | 54.4 | 39.2 | 33.7 | + | 5.2 | 9.49 | 213 | UKN | No | + | + |
| 62 | M | 26 | 7.10E+06 | No | 108 | 49.2 | 35 | 49.6 | 41.3 | 20 | + | 15.25 | 4.51 | 181 | UKN | No | + | + |
| 63 | F | 30 | 8.90E+06 | No | 32.1 | 27 | 14.5 | 60.8 | 44 | 24.8 | + | UKN | 3.63 | 85 | UKN | No | + | + |
| 64 | F | 30 | 4.00E+04 | No | 196 | 41.2 | 49.8 | 77.1 | 50.6 | 35 | + | UKN | 2.7 | 131 | UKN | No | + | + |
| 65 | F | 26 | 6.50E+07 | No | 25.8 | 81.3 | 64.4 | 51 | 44.4 | 22.2 | + | UKN | 2.54 | 132 | UKN | No | + | + |
| 66 | M | 35 | 1.20E+06 | No | 103.5 | 73.9 | 68.1 | 85.1 | 53.8 | 30.5 | + | UKN | 4.27 | 84 | 5.47 | No | + | + |
| 67 | M | 23 | 1.60E+08 | No | 57.2 | 39.1 | 26.2 | 79.6 | 42.3 | 27.4 | + | UKN | 9.08 | 260 | 1.93 | No | + | + |
| 68 | M | 33 | 9.70E+08 | No | 43.1 | 116.1 | 37.9 | 68.8 | 46.1 | 25.6 | + | UKN | 4.8 | 139 | UKN | No | + | + |
| 69 | M | 37 | 1.90E+06 | No | 197.4 | 123.6 | 35.9 | 96.7 | 41.1 | 32.1 | + | UKN | 4.59 | 121 | UKN | No | + | + |
| 70 | F | 29 | 1.10E+05 | No | 115.2 | 65.8 | 29.3 | 47.8 | 49.8 | 31.8 | + | UKN | 5.85 | 136 | UKN | No | + | + |
| 71 | M | 25 | 1.60E+06 | No | 54.3 | 110.2 | 38.1 | 53.7 | 51.4 | 27.8 | + | UKN | 3 | 91 | UKN | No | + | + |
| 72 | F | 20 | 3.70E+05 | No | 92.8 | 84.8 | 99.8 | 46.7 | 45.7 | 32 | + | UKN | 4.21 | 211 | UKN | No | + | UKN |
| 73 | M | 32 | 4.20E+08 | No | 123.5 | 92.3 | 108.6 | 89 | 41.5 | 30.7 | + | UKN | 4.3 | 116 | 107.16 | No | + | + |
| 74 | F | 21 | 1.40E+07 | No | 35.6 | 117 | 21.6 | 77.7 | 46.2 | 22.2 | UKN | UKN | 4.6 | 108 | UKN | No | + | UKN |
| 75 | M | 29 | 9.40E+06 | No | 121.8 | 176.2 | 51 | 48 | 46.8 | 23.5 | + | UKN | 4.66 | 82 | UKN | No | + | + |
| 76 | M | 22 | 3.30E+06 | No | 282.8 | 130.9 | 71.4 | 45.3 | 43.4 | 24 | + | UKN | 8.74 | 108 | UKN | No | + | + |
| 77 | M | 25 | 2.80E+06 | No | 66.1 | 241.9 | 33.5 | 75 | 48.8 | 25.1 | + | 15.38 | 4.74 | 96 | 6.13 | No | + | + |
| 78 | M | 40 | 2.50E+07 | No | 243.9 | 59.7 | 118.7 | 84.8 | 44.3 | 21.4 | + | UKN | 6.83 | 206 | 9.17 | No | + | + |

Num: patients number; ADJ THER: adjuvant therapy; ALT: alanine aminotransferase; AST: aspartate aminotransferase; GTP: glutamyl transpeptidase; ALP: alkaline phosphatase; ALB: albumin; GLU: globulin; WBC: leucocytes; PLT: platelets; AFP: α-fetoprotein. F: female; M: male; +: positive; UKN: unknown. HBV-DNA unit: copy/ml.

**Supplementary Table S3.** The serum levels of HCMV-encoding miRNAs in the training set.

| **Gene name** | **IFNα-RES**  Mean ± SEM (n=10), fmol/L | **IFNα-NRS**  Mean ± SEM (n=10), fmol/L | **IFNα-RES/ IFNα-NRS**  fold change | **P value**  *t*-test |
| --- | --- | --- | --- | --- |
| hcmv-mir-US4 | 150.79±21.38 | 806.05±93.43 | 5.34 | 2.13×10^-6^ |
| hcmv-mir-UL148D | 40.24±8.25 | 140.12±32.39 | 3.38 | 1.31×10^-4^ |
| hcmv-mir-UL112 | 42.23±3.04 | 64.73±10.11 | 1.53 | 0.0472 |
| hcmv-mir-US25-2-5p | 17.43±2.64 | 23.75±1.32 | 1.36 | 0.0461 |
| hcmv-mir-US5-1 | 4.32±0.56 | 3.45±0.42 | 0.79 | 0.1272 |
| hcmv-mir-US5-2 | 8.26±1.11 | 11.92±1.33 | 1.44 | 0.0497 |
| hcmv-mir-UL36 | 3.59±0.94 | 2.88±0.57 | 0.82 | 0.5273 |
| hcmv-mir-UL22a | 0.05.±0.003 | 0.03±0.001 | 0.64 | 0.0102 |
| hcmv-mir-UL70-5p | 0.26±0.02 | 0.18±0.04 | 0.69 | 0.0165 |
| hcmv-mir-US25-1 | 0.04±0.01 | 0.05±0.003 | 1.25 | 0.3742 |
| hcmv-mir-US25-2-3p | 0.01±0.002 | 0.02±0.002 | 1.42 | 0.2943 |
| hcmv-mir-33-3p | 0.43±0.03 | 0.39±0.05 | 0.91 | 0.6321 |
| hcmv-mir-33-5p | 0.37±0.02 | 0.28±0.03 | 0.76 | 0.1914 |

IFNα-RES: IFNα-responsive group; IFNα-NRS: IFNα-non-responsive group.

**Supplementary Table S4.** The serum levels of HCMV-encoding miRNAs in the validation set.

| **Gene name** | **IFNα-RES**  Mean ± SEM (n=18), fmol/L | **IFNα-NRS**  Mean ± SEM (n=10), fmol/L | **IFNα-RES/ IFNα-NRS**  fold change | **P value**  *t*-test |
| --- | --- | --- | --- | --- |
| hcmv-mir-US4 | 163.59±12.82 | 830.69±58.43 | 5.08 | 6.66×10^-13^ |
| hcmv-mir-UL148D | 38.02±4.15 | 133.44±9.72 | 3.51 | 7.26×10^-6^ |
| hcmv-mir-UL112 | 48.71±7.08 | 41.44±3.85 | 0.85 | 0.3738 |
| hcmv-mir-US5-2 | 8.79±0.98 | 11.95±0.91 | 1.36 | 0.1482 |
| hcmv-mir-US25-2-5p | 19.22±2.73 | 19.37±4.24 | 1.01 | 0.9749 |

IFNα-RES: IFNα-responsive group; IFNα-NRS: IFNα-non-responsive group.

**Supplementary Table S5.** Clinic characteristics of CHB patients.

| Characteristics | | IFNα-responsive (n=10) | IFNα-non-responsive (n=10) | p-value |
| --- | --- | --- | --- | --- |
| Age (years) | | 36.6±2.98 | 38.3±3.62 | 0.747^a^ |
| Sex | Male | 7 | 6 | 0.219^b^ |
|  | Female | 3 | 4 |  |
| HBV-DNA(10^7^ copies/ml) | | 2.27±1.67 | 1.52±0.93 | 0.713^a^ |
| Adjuvant therapy | Yes | 1 | 2 | 0392 ^b^ |
|  | No | 9 | 8 |  |
| Response after treatment | HBV-DNA<500 copies/ml | 7 | 0 |  |
|  | HBV-DNA level decreases > 90% after IFNα therapy | 10 | 0 |  |

a, student-t test; b, two-sided χ2 test.

HBV-DNA < 500 copy/ml or HBV-DNA level after IFNα therapy/HBV-DNA level before IFNα therapy <0.1 was considered as IFNα effective.


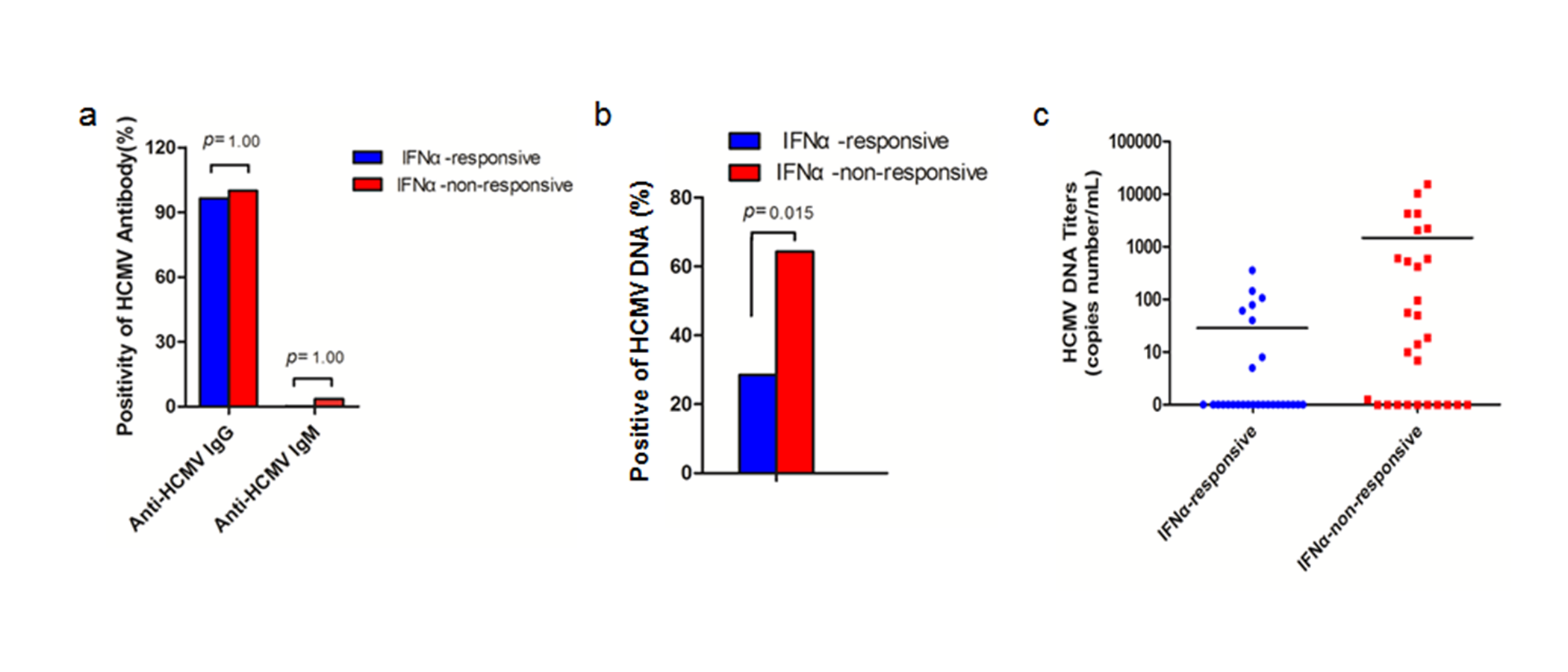


**Supplementary Figure S1.** Human cytomegalovirus (HCMV)-positive status in IFNα-RES and IFNα-NRS CHB patients (n=28 each). (a) The seropositivity rates of anti-HCMV IgG and anti-HCMV IgM are shown in IFNα-RES and IFNα-NRS CHB patients. P values were calculated by the χ^2^ test. (b) Positive rate of HCMV DNA in the IFNα-RES group and IFNα-NRS group; *P* values were calculated by the χ^2^ test.(c) The HCMV virus titer was calculated in IFNα-RES group and IFNα-NRS group (*P* = 0.017); *P* values were calculated by a 2-sided Student *t*-test.


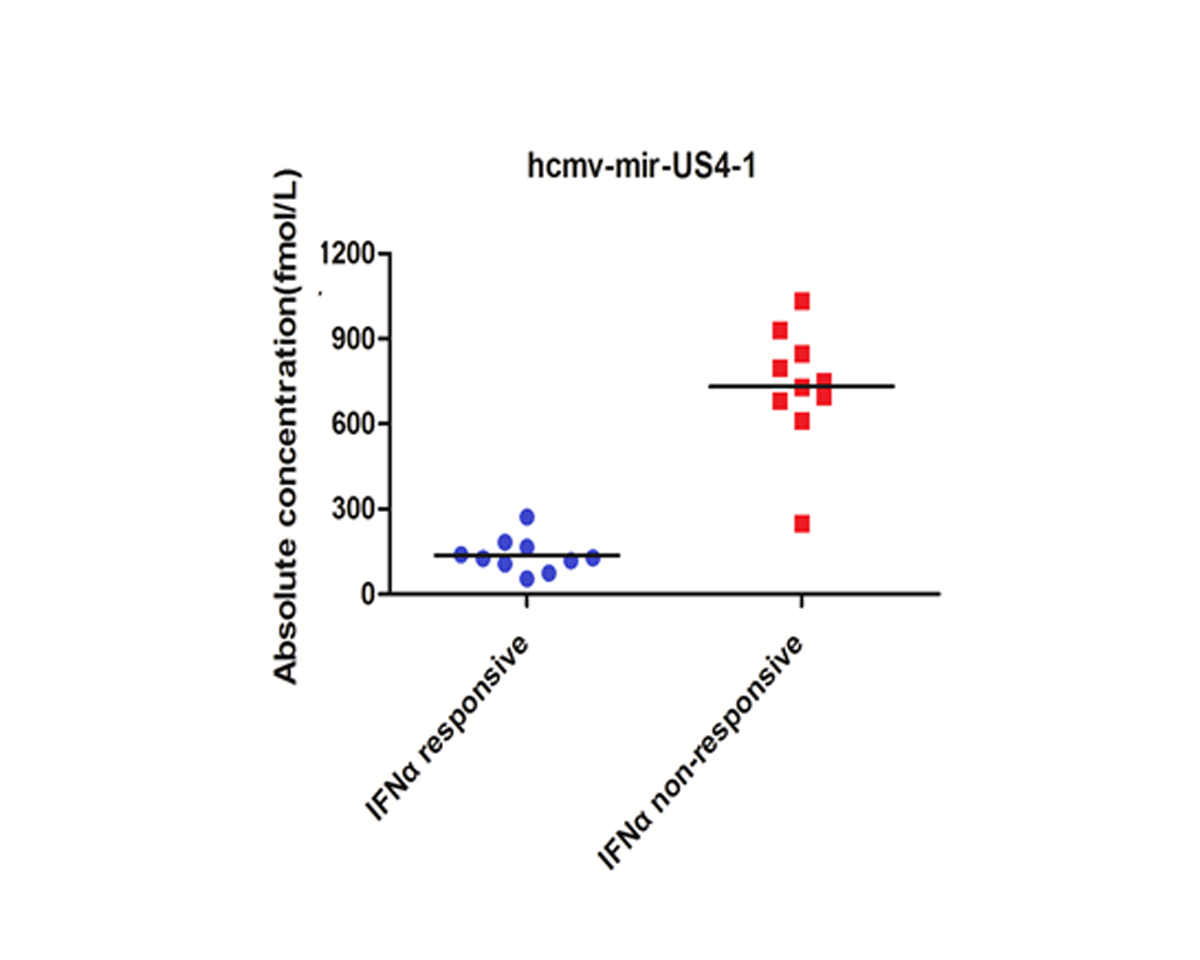


**Supplementary Figure S2.** Absolute level of hcmv-mir-US4-1 in IFNα-RES and IFNα-NRS CHB patients (n=10 each) after IFNα treatment.
